# Mechanisms of action of an implementation intervention in stroke rehabilitation: a qualitative interview study

**DOI:** 10.1186/s12913-016-1793-8

**Published:** 2016-09-30

**Authors:** Louise A. Connell, Naoimh E. McMahon, Sarah F. Tyson, Caroline L. Watkins, Janice J. Eng

**Affiliations:** 1 College of Health & Wellbeing, University of Central Lancashire, Preston, PR1 2HE UK; 2 Stroke & Vascular Research Centre, School of Nursing, Midwifery & Social Work, Jean McFarlane Building, University of Manchester, Oxford Rd, Manchester, M13 9PL UK; 3 Department of Physical Therapy, University of British Columbia, 212-2177 Wesbrook Mall, Vancouver, BC V6T 1Z3 Canada

**Keywords:** Stroke, Implementation, Behaviour change, Mechanisms of action, Qualitative design

## Abstract

**Background:**

Despite best evidence demonstrating the effectiveness of increased intensity of exercise after stroke, current levels of therapy continue to be below those required to optimise motor recovery. We developed and tested an implementation intervention that aims to increase arm exercise in stroke rehabilitation. The aim of this study was to illustrate the use of a behaviour change framework, the Behaviour Change Wheel, to identify the mechanisms of action that explain how the intervention produced change.

**Methods:**

We implemented the intervention at three stroke rehabilitation units in the United Kingdom. A purposive sample of therapy team members were recruited to participate in semi-structured interviews to explore their perceptions of how the intervention produced change at their work place. Audio recordings were transcribed and imported into NVivo 10 for content analysis. Two coders separately analysed the transcripts and coded emergent mechanisms. Mechanisms were categorised using the Theoretical Domains Framework (TDF) (an extension of the Capability, Opportunity, Motivation and Behaviour model (COM-B) at the hub of the Behaviour Change Wheel).

**Results:**

We identified five main mechanisms of action: ‘social/professional role and identity’, ‘intentions’, ‘reinforcement’, ‘behavioural regulation’ and ‘beliefs about consequences’. At the outset, participants viewed the research team as an external influence for whom they endeavoured to complete the study activities. The study design, with a focus on implementation in real world settings, influenced participants’ intentions to implement the intervention components. Monthly meetings between the research and therapy teams were central to the intervention and acted as prompt or reminder to sustain implementation. The phased approach to introducing and implementing intervention components influenced participants’ beliefs about the feasibility of implementation.

**Conclusions:**

The Behaviour Change Wheel, and in particular the Theoretical Domains Framework, were used to investigate mechanisms of action of an implementation intervention. This approach allowed for consideration of a range of possible mechanisms, and allowed us to categorise these mechanisms using an established behaviour change framework. Identification of the mechanisms of action, following testing of the intervention in a number of settings, has resulted in a refined and more robust intervention programme theory for future testing.

**Electronic supplementary material:**

The online version of this article (doi:10.1186/s12913-016-1793-8) contains supplementary material, which is available to authorized users.

## Background

The time it takes for research to translate into clinical practice is unacceptably long [1]. Systematic reviews and clinical guidelines have been devised in efforts to bridge this evidence-practice gap. However, such publications alone are not enough to initiate and sustain a change in the day-to-day practices of clinicians [2], and the underpinning evidence is often criticised for not being reflective of how interventions are delivered in the real clinical context. There remains a lack of understanding about contextual factors influencing stoke rehabilitation practice. For example, despite best evidence demonstrating the effectiveness of increased intensity of exercise after stroke, current levels of therapy continue to be below those required to optimise motor recovery [3, 4]. Several therapy interventions have been developed to address this problem with respect to recovery of the arm after stroke but important aspects of these interventions are often modified when implemented in routine care [5]. Thus the extent to which they achieve their aim of increasing intensity of exercise in real world rehabilitation settings is unclear. We have developed an implementation intervention, underpinned by implementation theory, that aims to increase arm exercise in stroke rehabilitation by changing the behaviour of therapists. The intervention is underpinned by formative research on an evidence-based arm rehabilitation intervention, the Graded Repetitive Arm Supplementary Programme (GRASP) [6]. GRASP is a self-directed hand and arm exercise programme which is taught and monitored by a therapist, but carried out by the patient with the support of their family/carer where possible. GRASP is not meant to replace existing therapy services, rather to augment current therapy, adding opportunities for more practice. Similar to existing therapy interventions, GRASP involves a complex implementation chain influenced by interactions between patients, therapists and the wider rehabilitation environment. The fidelity to the intervention in clinical settings had been shown to be variable [5].

Development of the implementation intervention was guided by the Behaviour Change Wheel (BCW) [7], the details of which have been reported elsewhere [8]. In brief, we worked collaboratively with a therapy team at a local stroke unit and other stakeholders, establishing an active partnership in an intervention development group. Structured discussions were undertaken to (i) understand the problem (i.e. intensity of arm exercise in the stroke rehabilitation unit) and to identify target behaviours that would be amenable to change, (ii) design intervention components that could change behaviours through established behaviour change techniques and (iii) pilot and refine the developed intervention components. The resulting intervention was called PRACTISE (Promoting Recovery of the Arm: Clinical Tools for Intensive Stroke Exercise). It consists of face-to-face meetings between the research team and therapy teams, and materials to aid implementation using established behaviour change techniques, a novel aspect of this implementation study compared with many previous rehabilitation studies. PRACTISE aims to address four target behaviours of therapists: (i) screening patients for suitability for supplementary self-directed arm exercise, (ii) provision of arm exercises, (iii) involving family/carers in assisting with exercises and (iv) monitoring and reviewing exercises.

Our original programme theory for PRACTISE was developed using the Capability, Opportunity, Motivation and Behaviour model (COM-B) at the hub of the BCW and is summarised in Table 1. The behaviour change techniques underpinning the intervention components were identified using the Behaviour Change Technique Taxonomy v1 (numbers shown correspond with how the 93 techniques are clustered into 16 groups in the taxonomy) [9].

**Table 1:** Original proposed PRACTISE intervention components and mechanisms of action using the behaviour change wheel; capability, motivation, behaviour model; and BCT taxonomy (v1)

Intervention components	Determinant of behaviour from COM-B		Behaviour change techniques from BCTTv1^a^ [13]
Screening tool PRACTISE exercise pack	Physical Opportunity	Due to time constraints more efficient ways of performing the target behaviours were needed	4.1 Instruction on how to perform the behaviour 3.2 Social support (practical)
Team meetings Audit tool	Social Opportunity	Getting upper limb rehabilitation higher up on the agenda was needed through managerial support and team engagement	1.2 Problem solving 1.4 Action planning 1.9 Commitment 2.3 Self-monitoring of behaviour

BCTTv1: Behaviour Change Technique Taxonomy v1

^a^Behaviour change techniques are numbered based on the Behaviour Change Technique Taxonomy

We intended to influence physical and social opportunity to perform the target behaviours by making it easier for therapy teams to provide supplementary self-directed arm exercises to patients, and by making the provision of arm exercises a priority in the stroke rehabilitation unit. Monthly meetings between the research team and therapy teams provided an opportunity to discuss emergent barriers to implementation and to identify context appropriate solutions, which were intended to maximise commitment to the study and implementation of the intervention. Materials to support implementation included a screening tool and exercise pack to make it quicker and easier to provide exercises to suitable patients. The audit tool provided a method of self-monitoring for performance of the target behaviours at a service level. We anticipated that this would work in two ways. Firstly, as a weekly reminder about what needed to be done. Secondly, if there was a discrepancy between what the teams proposed to do and what they actually did, this would be highlighted and act as an incentive to improve for the following week.

We implemented PRACTISE in two additional stroke units to explore the feasibility and acceptability of the intervention to staff and patients, along with examining the ways in which the intervention facilitated change. To develop effective implementation interventions researchers are being urged not just to establish that an intervention works, but also to identify and explain the specific ways in which it works i.e. the mechanisms of action [10]. Mechanisms have been defined as “hidden but real” and as elements “of reasoning and reactions of agents in regard to the resources available in a given context” [11]. The term mechanism has been conceptualised and operationalised differently in the literature, the most well-known approach perhaps being the context-mechanism-outcome configurations used in realist evaluation [12]. For the purposes of our study we considered mechanisms to be those less observable “things” that happen in the black box between behaviour change techniques and components, and observed outcomes.

Although work is ongoing to establish a method for linking behaviour change techniques to mechanisms of action [13] there is not yet an established approach for identifying and reporting the mechanisms of action of behaviour change or the components of implementation interventions. The aim of this study was to use the BCW to identify mechanisms of action and provide a rich explanation as to how our implementation intervention supported change at a site level. We consider the emergent mechanisms in light of our original programme theory to present a refined intervention programme theory for future testing.

## Methods

### Implementation of the intervention

The characteristics of the participating sites are shown in Table 2. A detailed report on the implementation of the intervention across the three sites (Sites A, B & C), and staff and patients’ perceptions of the feasibility and acceptability of the intervention will be reported in detail separately. The extent to which the target behaviours changed in the three stroke rehabilitation units over the course of the study is shown in Table 3.

**Table 2:** Characteristics of participating sites

Site information	Site A	Site B	Site C
Organisation	General hospital	General hospital	General hospital
Number of stroke beds	23	24	24
Patients admitted from	Emergency department	Hyper-acute stroke ward	Hyper-acute stroke ward
Average length of stay	18.5 days	Missing	23 days
Weekday therapy input	Target of 45 mins therapy per discipline per day	Target of 45 mins of each therapy per day	Target of 45 mins of each therapy per day
Weekend therapy input	Reduced Saturday service (prioritise chest physiotherapy and new patients) No service on Sundays	Reduced Saturday service (prioritise chest physiotherapy and new patients) No service on Sundays	None routinely
Staffing (WTE, when full)	PT: 6.0 OT: 6.0 Assistants: 3.0	PT: 3.8 OT: 4.0 Assistants: 4.5	PT: 3.1 OT: 2.8 Assistants: 1.7

**Table 3:** Change in target behaviours

Target behaviour	Site A	Site B	Site C
Screening patients for suitability for supplementary self-directed arm exercise	✓	✓	✓
Provision of arm exercises	✓	✓	✓
Involving family/carers in assisting with exercises	O	✘	✓
Monitoring and reviewing exercises	✘	✘	✓

✓ = most change in performance

O = implemented alternative change than proposed in the intervention

✘ = least change in performance

PRACTISE resulted in change in at least two of the four target behaviours at each site. In site A, therapy assistants provided assistance and supervision to patients while performing their upper limb exercises, rather than family/carers. At Site B, resignation of senior staff and ongoing service re-organisation limited the extent to which we could progress through the phased implementation approach in the six month study period. Site C, acted as the development site for the intervention and therefore had a longer “embedding period”. Changes in all target behaviours were achieved at this site and were sustained as a result of including upper limb therapy practice in an internal departmental audit. The least change was seen in monitoring and progressing of exercises across the three stroke units due to a short length of stay.

### Study design

Qualitative interview study.

### Theoretical framework

We used a directed content analysis approach with behaviour change theory used as guidance for initial codes [14].

### Participant selection

All physiotherapists, occupational therapists, therapy managers and therapy assistants in the participating sites were involved in the embedding of PRACTISE. A purposive sample of these participants were invited to take part in interviews to explore their perceptions of how the intervention produced change, or not, at their site during the study. The rationale for conducting interviews over the six month study period was to learn about the processes of change, and how these may have developed from the beginning to the end of the study. Between two and three participants were interviewed during each visit based on their availability. Participants were only interviewed once over the course of the study. On average, interviews took approximately 45 min. We ceased recruiting therapy team members once data saturation was reached (i.e. no new themes were emerging) or all members of the therapy team had participated.

### Setting

We used a six month phased approach to implementation in three stroke rehabilitation units in the North West of England (the stroke unit where the intervention was developed and two additional stroke units). Implementation was guided by the target behaviours (i.e. starting with screening of patients before progressing to provision of arm exercises) and commenced at Sites A and B in October 2014. Site C acted as the development site for the intervention from December 2013 to June 2014.

### Data collection

Semi-structured face-to-face interviews were conducted to explore therapists’ perceptions of how the intervention produced, or failed to produce, change were conducted by LC and NM on site in quiet spaces and at convenient times for the interviewees. Where possible, interviews were conducted in private offices, but sometimes they were conducted in quiet corners of public spaces, e.g. the hospital canteen due to space limitations. Normalisation Process Theory (NPT) was used to develop an interview guide (Additional file 1). NPT is a sociological toolkit to understand the work that is done to implement and embed complex interventions in healthcare settings [15]. Particular emphasis was placed on probing questions that encouraged participants to reflect on what supported change throughout the stages of implementation. Interviews were audio-recorded and all participants provided written informed consent prior to the interview. Field notes were made after each site visit to document the following: observations, the content of monthly meetings; ad hoc discussions with therapists; additional contacts (e.g. email) between meetings and reasons for these; and informal discussions on the progress of the study by therapists and managers. These data were summarised at the end of data collection period to provide more detailed insight into the process of implementation and possible mechanisms, providing a method of triangulation.

### Data analysis

Audio recordings were transcribed and imported into NVivo 10 for content analysis. Interview transcripts were coded by LC and NM using predetermined codes based on the Theoretical Domains Framework (TDF) [16]. The TDF is an extension of the Capability, Opportunity and Motivation model (COM-B) at the hub of the BCW. Codes were compared between researchers and non-fitting items discussed. An agreement was reached on where the mechanisms fit within the TDF, with any further points of contention discussed with all the authors and agreement sought. Emergent mechanisms were discussed with study participants to ensure that the data had been accurately interpreted and to provide opportunity for clarification of preliminary findings. The final coding process involved free coding of text where participants provided rich and insightful reflections as to how and why the intervention produced change.

#### Synthesis of Results to produce mechanisms of action for PRACTISE

Following the final coding process, the research team met to synthesise the results by listing the intervention components and to relate these to the findings from the perceived mechanisms of action. Discrepancies between the determinants of behaviour as assigned a priori in the development stage using the COM-B model, and possible mechanisms of action as identified by the TDF were discussed and agreement made about how the intervention is understood to work. Issues with this will be considered in the discussion.

### Research team

LC and NM are both chartered physiotherapists with experience of qualitative research methods. Both hold full-time research positions at a UK university working on a National Institute for Health Research (NIHR) funded project to develop a clinically feasible and structured upper limb exercise programme for use in National Health Service (NHS) stroke rehabilitation units. They built a relationship with the therapy teams at each of the sites throughout the study but were not known to the participants beforehand. The therapy teams were informed at the outset that the purpose of the research was to explore the feasibility of implementing practice change in stroke rehabilitation settings as opposed to demonstrating the effectiveness of a specific intervention. ST, CW and JE are stroke rehabilitation researchers. JE developed GRASP, the intervention from which this study emerged, and conducted the randomised trial confirming its effectiveness [6]. A directed approach to content analysis was undertaken as it makes explicit that the research team had experience of implementation theories and were not working from a naïve perspective, and fitted with the aim of the study to use existing theory (the TDF) to try and unpick mechanisms of action of the intervention.

## Results

### Characteristics of interview participants

Twenty-three therapy team members were interviewed: 8 physiotherapists, 11 occupational therapists and four therapy assistants (Table 4). Six participants had been qualified for five years or less and 10 participants had more than five years’ experience specifically working with people with stroke. Of the qualified staff, two were junior (NHS band 5), 14 were senior (NHS band 6 or 7), and 3 were team leads/therapy manager. Only one staff member was male. The breakdown of demographic by participant, or identifiers within the included quotes, has not been included to protect the anonymity of the participants.

**Table 4:** Interview participants across sites

Site	Total	PT	OT	Assistant
A	12	5	6	1
B	6	2	3	1
C	5	1	2	2

### Mechanisms of action

Using the TDF, we identified five mechanisms that could explain how, or why, PRACTISE produced the observed changes. These included: ‘social/professional role and identity’; ‘intentions’; ‘reinforcement’; ‘behavioural regulation’, and ‘beliefs about consequences’. Definitions of these TDF mechanisms are provided below. For some emergent mechanisms, clear links with specific components of the intervention, or behaviour change techniques could be identified but quite often, participants discussed how their involvement in the study as a whole resulted in change at their site.

### Social/professional role and identity

This domain relates to a coherent set of behaviours and displayed personal qualities of an individual in a social or work setting. Participants accredited their team’s engagement with the study to two factors relating to professional roles. Firstly, they viewed the research team as an external influence for whom they wanted to ensure the required work was completed. This links to the constructs of professional credibility and identity within the TDF. Secondly, they valued their relationship with the university, which gave an impetus to ensure they delivered the required work. The social identity, and how therapists related to the research team influenced their behavior.

**Site A, PT04:** “Even if you had someone sort of driving it forward within the team, I don’t know whether it has quite the same effect as an external force that gives you that sort of…“yeah I really should do that before they come in”…But that’s no bad thing actually until it’s at the point where it’s embedded.”

**Site C, OT11:** “For me personally, I think there was a big impetus to do it because it was linked with UCLan initially. You kind of have something that you’re aiming towards.”

### Intentions

Intentions relate to a conscious decision to perform a behaviour or a resolve to act in a certain way. Participants accredited their intent to engage with the study to its design. At the outset, we stressed that the purpose of the study was to test the feasibility of implementing PRACTISE at their work setting and that all feedback or suggested revisions would be welcome. This meant therapists did not feel threatened and were willing to move from contemplation and preparation to action. This theme emerged particularly strongly at Site B where the therapy team were implementing PRACTISE during a service re-organisation, and hence had difficulties performing the target behaviours. When therapy teams were reassured that capturing all of these experiences and challenges was worthwhile for the research, they felt under less pressure to perform all target behaviours consistently, and as a consequence persevered with the study processes. Emphasising that PRACTISE could, and should, fit with ‘real life working’ seemed to resonate with participants and was very much in contrast to their past research experiences.

**Site B, OT09:** “I think because there’s been so many problems in the team, there was talk about people wanting to withdraw but then the meeting that I went to where LC said “we know it’s not been very good, and we know it’s all been tricky for you, don’t worry about it and that’s useful information to us”. I think that really helped and everyone was like “oh, that’s alright then” because it felt like we were failing before and it was like a stress that we couldn’t manage and that we weren’t doing what was asked of us but then with LC saying that everyone was like “oh brill”…”

**Site A, OT06:** “I just think that it’s been a really nice relaxed project to be involved in. We’ve never felt pressurised into getting the results and suchlike…Sometimes when you’re involved in research it’s…you’ve just got to get the numbers in and it becomes a real sort of turn off in some respects.”

At the development site, Site C, upper limb therapy input was used for the team’s internal annual audit, which acted as a driving force to sustain implementation even after the research team’s involvement had come to an end.

**Site C, PT08:** “You guys obviously took a step back so that was less of a drive really to keep it going, but then because the project was linked with our departmental audit, that then gave us another deadline that we had to work towards…So myself and one of the OT’s had to get our act together again, to gather that data for a slightly different reason but that has made the rest of the team, and now the new staff that have come in, more aware of that process and it has become embedded again within our practice I would say.”

### Reinforcement

Reinforcement relates to increasing the probability of a response by arranging a dependent relationship between the response and a given stimulus. For the PRACTISE intervention, the active involvement of the researchers and the regular team meetings provided reinforcement to perform the target behaviours, and meant that there was recognition amongst peers if behaviours were performed, and conversely negative consequences of reporting that behaviours weren’t being undertaken.

**Site A, PT04:** “As I said, it’s been good that you guys have been coming because I think it’s kept us thinking about it and it’s also moved it forward…”

**Site C, OT11:** “I do think it’s been quite valuable to have consistent input from the people introducing the treatment activities; not just to amend it or whatever but to keep the momentum going.”

The challenge of maintaining momentum when implementing new treatment approaches was highlighted in the interviews. Participants discussed past experiences of colleagues who have returned to work after attending Continuing Professional Development (CPD) events. New ideas from these events were often very well received when first introduced but with time tended to fall by the wayside.

**Site A, PT02:** “From experience, things are very hot when they’re new and then kind of tail off. And every now and again, someone will remember it and try and pick it up but I think this (regular meetings) seems to be quite a nice refresher.”

### Behavioural regulation

Behavioural regulation is anything aimed at changing objectively measured actions. The PRACTISE intervention relates to the constructs of self-monitoring and action planning. The purpose of the audit tool was specifically to facilitate self-monitoring performance of target behaviours. Participants confirmed that the audit tool in weekly meetings acted as a reminder to keep up the PRACTISE activities. However, they viewed the tool more as research data than as a method of monitoring overall service performance. Site C was an exception, as they were using the data collected to conduct an internal audit in their department. Therapists also discussed how the team meetings acted as a prompt to plan who would be responsible for each of the target behaviours for each patient.

**Site A, PT03:** “Yeah and I kind of feel like, if I was to work anywhere else I’d find something similar useful so…each week we go “OK, who needs a PRACTISE programme?” and having our tick boxes, because otherwise I think it’s very easy to forget about these tools.”

**Site A, PT04:** “I can see where it does sort of help. You know it’s nice to look back and it’s nice to go “OK you have done that”…but I don’t think we’ve made any attempt to really look at it as a team. I don’t think that’s what’s driven us forward which is why I don’t know whether it would make a difference if we continued it or not, or whether we would see it as just another bit of paperwork that needs looking at and doing.”

### Beliefs about consequences

This domain relates to acceptance of the truth, reality, or validity about outcomes of a behaviour in a given situation. In this instance, beliefs about consequences refers to the consequences of implementing PRACTISE for the therapy teams, rather than the consequences for patients. At the outset, therapists were understandably concerned about the feasibility of implementing something new with already constrained resources. However, as the study progressed, therapists’ attitudes towards the value of the intervention seemed to change whereby it was no longer seen as an added burden but an integral part of their therapy that brought reward.

**Site A, PT05:** “I think it’s definitely been worthwhile, I think it’s really changed what we’re doing on the unit… It’s something additional that we don’t necessarily have time to focus on in therapy sessions, so it gives an extra opportunity for more therapy throughout the day…I think despite all those kind of initial thoughts it’s…now we’re at the point where it’s just part of what we do.”

**Site A, PT03:** “At first I think it was difficult because it was like an extra thing to do so you had your own treatment plan of what you wanted to do with a patient then you’re like oh, I have to do this PRACTISE as well so to begin with I kind of saw it as a separate thing…I don’t now, I must say it’s given me loads of good little ideas with for exercises with them. I’d say that I definitely incorporate it more into the therapy session and as opposed to an add-on.”

### PRACTISE intervention components and mechanisms of action

A refined list of the PRACTISE intervention components and mechanisms of action which form the refined programme theory are presented in Table 5. More detailed description on the components of the intervention are provided with a related expansion in the behaviour change techniques delivered through these components. The TDF mechanisms of action are presented along with their relationship to the COM-B model categories. The content has been organised to be readable and show some connections between actions, techniques and mechanisms. However, in reality these overlap and mechanisms can act as precursors or successors to each other. Although ‘environmental context and resources’ did not emerge as a particularly strong mechanism in the interviews we have included it here as a potential explanation as to how techniques such as ‘adding objects to the environment’ may produce change. It is perhaps the case, that physical restructuring of an environment is not a mechanism in itself, but that it works by bringing about a change in other mechanisms: for example intentions. This will be considered further in the discussion. Conversely, the mechanism ‘beliefs about consequences’ was not attributable to individual intervention components or activities but instead emerged as a reflection on the study process as a whole. It has therefore not been included in Table 5.

**Table 5:** Refined PRACTISE intervention components and and BCT taxonomy (v1); mechanisms of action using the behaviour change wheel; capability, motivation, behaviour model; and TDF

Monthly meetings between research team and therapy teams			
Intervention components	Behaviour change techniques from BCTTv1 [13]	Determinant of behaviour from COM-B	Mechanisms of action from TDF
Monthly meetings between research and therapy teams	7.1 Prompts/cues	Automatic motivation	Reinforcement Beliefs about consequences
Research team from local university	10.5 Social incentive	Reflective motivation	Social/professional role and identify
Phased approach focussing on feasibility of implementation	4.1 Instruction on how to perform the behaviour 8.7 Graded tasks	Reflective motivation	Intentions
Identified barriers to performing target behaviours and developed strategies to overcome them	1.2 Problem solving 1.4 Action planning 3.2 Social support (practical) 12.5. Adding objects to the environment	Reflective motivation Physical opportunity	Intentions Behavioural regulation
Intervention components	Behaviour change techniques from BCTTv1 [13]	Source of behaviour from COM-B	Mechanisms of action from TDF
Screening tool & exercise pack			
Materials provided to assist performance of the target behaviours	12.5 Adding objects to the environment	Physical opportunity	Environmental context and resources
Audit tool			
Therapy teams asked to document performance of the target behaviours and provide feedback by research team	2.2 Feedback on behaviour 2.3 Self-monitoring of behaviour 7.1 Prompts/cues	Psychological capability	Behavioural regulation

*BCTTv1* behaviour change technique taxonomy v1, *BCW* behaviour change wheel, *TDF* theoretical domains framework

## Discussion

We have demonstrated use of the Behaviour Change Wheel to identify five possible mechanisms of action of an implementation intervention. These included ‘social/professional role and identity’, ‘intentions’, ‘reinforcement’, ‘behavioural regulation’ and ‘beliefs about consequences’. In the original intervention development, we hypothesised that implementation of the intervention would occur through changes in physical and social opportunity. However, the emergent mechanisms most often related to reflective and automatic motivation (TDF domains of ‘social/professional role and identity’, ‘intentions’, ‘reinforcement’, and ‘beliefs about consequences). At the outset, the therapy teams’ motivation to engage in the study activities was attributed to the monthly visits with the research team to discuss progress, which motivated them to complete the study activities as much as possible in the interim periods. The phased approach to implementation and the focus on feasibility sustained motivation throughout. Furthermore, a collaborative working relationship with the research team that encouraged teams to provide feedback on the intervention, and how it could be refined or revised, gave therapists’ autonomy to adapt intervention components to fit with their local context. This contrasted with their prior experiences of implementing strict research trial protocols. Once the therapy teams perceived the intervention to be part of their routine work, and of some value, motivation was driven less by a feeling of having to implement the intervention and more by wanting to implement it. This fits with other stroke rehabilitation implementation research, which highlights the need for active management strategies and close collaboration with stakeholders [2].

The importance of exploring and reporting the mechanisms of action of interventions has been highlighted in methodological and reporting guidance including the Template for Intervention Description and Replication (TIDieR) [17] and the Medical Research Council guidance for process evaluations [10]. However, as yet there are few examples of how to operationalise this in implementation research. To date, the TDF has been used to (i) explore barriers and facilitators to performing target behaviours e.g. [18, 19], (ii) guide intervention development [20–22] and (iii) describe intervention content both prior to implementation and through retrospective analysis [23–25]. There are limited examples where this framework has been used to unpick the mechanisms of action of components of interventions. Where examples do exist, identification of the mechanisms of action is often oriented around hypothesized or expected mechanisms of action by mapping behaviour change techniques to domains of the TDF, rather than to qualitative analysis of mechanisms of action as experienced by the recipients of interventions [23, 26] thus limiting comparisons that can be made with our study.

### Methodological considerations

In this study we set out to identify mechanisms of action that supported change. This is not to present an overly positive picture of the intervention, or to ignore explanations as to why some changes did not occur. Barriers to change often related to the feasibility of performing target behaviours within contextual constraints. Therefore, by developing a thorough understanding of the mechanisms that promoted change, it may be possible to identify prerequisite contextual conditions that allow these mechanisms to be activated or thrive.

The TDF enabled categorising of the mechanisms of action, which had both strengths and limitations. It gave more detail than the COM-B, as it is an extension of this model, though arguably the definitions are more obtuse. Although definitions for the domains of the TDF are provided, it was not always easy to determine in which categories the emergent mechanisms would be best placed. Some of the terminology used in the TDF was found to be confusing and jargon heavy, which may not be helpful to some users of the research e.g. clinicians. This is perhaps an irony within implementation research, that the frameworks in themselves are not always user friendly. In addition, there is an underlying assumption that the mechanisms are static, whereas in reality they may be different for different people and across different contexts. The extent to which identified mechanisms were linked was also unclear. For example, it would seem plausible that the mechanisms under ‘social/professional role and identify’ could be considered antecedents to changes in ‘intentions’.

As mentioned in the results section, it was difficult to determine whether discussions around ‘environmental context and resources’ could be conceptualised as mechanisms in themselves, or whether adding objects to the environment (e.g. screening tool, exercise pack) triggered mechanisms such as ‘behavioural regulation’. When reviewing the domains of the TDF, it is clear that they include a mix of personal characteristics (e.g. social/professional role and identify), cognitive processes (e.g. intentions), responses or reactions to components and techniques (e.g. reinforcement) and physical changes (e.g. environmental context and resources). Further work may be needed to establish greater consistency about how the term ‘mechanism’ is conceptualised and operationalised using these frameworks.

### Strengths and limitations

A strength of this study is the use of an established framework to categorise and describe the mechanisms of action, which facilitated consistent definitions and terminology. In turn, this allows for improved understanding of the content of the intervention and the ways in which it may produce change. This will also allow for robust comparisons to be made across studies for further testing and development of behaviour change theory and frameworks. Conversely, this use of a directed content approach has some inherent limitations in that data is analysed with potential bias, and a desire to fit all responses to pre-determined categories even if the fit is not perfect. We attempted to minimise this through discussions with the research team and participants, to check interpretation of the results. However, a limitation of this study may be the reliability of the analysis process. Neither coder has undertaken formal training in use of the behaviour change framework or its taxonomy. Despite our best efforts to use terms and definitions accurately and consistently, some categories were broad and overlapping. Consequently, some mechanisms could be categorised in alternative ways. Furthermore, like all interview studies, we were reliant on participants’ willingness and ability to fully reflect on, and articulate, their experiences. The depth of reflection, and richness of explanation reported in the transcripts, was varied and meant that heavy reliance was put on the more detailed and insightful interview accounts to extract the mechanisms of action, rather than the transcripts describing more practical aspects of implementation. For this reason, it is important to clarify that the mechanisms presented here are intended to be useful explanations as to how the components of the intervention may produce change. Alternative mechanisms may have been present that were not captured in the interview transcripts, and it is likely that participants will experience different mechanisms and in different ways. This is a limitation of behaviour change taxonomies, which are grounded in psychological theory, with contextual aspects given little consideration. This contrasts with, for example, realist methodologies. It was clear in this study, that interventions work through different mechanisms in different environments, and it is not yet clear how to describe or account for this using the TDF.

LC and NM facilitated implementation at each site and also conducted the interviews. Participants may have been inclined to provide favourable responses to the interviewers’ questions (i.e. a social desirability bias [27]) but it was stressed throughout that the purpose of the study was to learn about the process of implementing the intervention to encourage participants to be candid in relaying their experiences. Participants were interviewed at different time points through the implementation process, and repeated interviews were not possible due to limiting time demands on the staff involved. However, it has been shown that recall of the processes involved in implementation can be limited [5] therefore interviewing people at different points along the implementation process was important.

## Conclusions

There is increasing emphasis being placed on establishing not only if an intervention works, but how it works. We have illustrated the use of the Behaviour Change Wheel, and in particular the use of the Theoretical Domains Framework, to investigate mechanisms of action of an implementation intervention. This approach allowed for consideration of a range of possible mechanisms, and allowed us to categorise these mechanisms using an established behaviour change framework. Identification of the mechanisms of action, following testing of the intervention in a number of settings, has resulted in a refined and more robust intervention programme theory for future testing.

## Additional file

Additional file 1: Appendix 1. Interview guide. Details of the interview guide used, developed using NPT. (DOCX 18 kb)

## Abbreviations

BCTTv1: Behaviour change technique taxonomy v1
BCW: Behaviour change wheel
COM-B: Capability opportunity motivation and behaviour
CPD: Continuing professional development
GRASP: Graded repetitive arm supplementary programme
NPT: Normalisation process theory
NIHR: National Institute for Health Research
NRES: National research ethics service
TDF: Theoretical domains framework
TiDIER: Template for intervention description and replication
PRACTISE: Promoting recovery of the arm: clinical tools for intensive exercise
