# Supplementary material for: Mechanisms of action of an implementation intervention in stroke rehabilitation: a qualitative interview study
Source: BMC Health Serv Res. 2016 Sep 30;16:534. doi: 10.1186/s12913-016-1793-8 (PMC5045623; doi:10.1186/s12913-016-1793-8)
Supplement: Additional file 1: — Appendix 1. Interview guide. Details of the interview guide used, developed using NPT. (DOCX 18 kb) [file 12913_2016_1793_MOESM1_ESM.docx]

**INTRODUCTORY QUESTIONS**

- Can you tell us about your role in stroke rehabilitation here at _________?
- What is the aim of the PRACTISE project?
- Can you describe in your own words what the PRACTISE intervention is?

**PROCESSES OF IMPLEMENTING GRASP**

**Coherence**

- Do you think the purpose of the project was clearly explained in the project set-up meetings?
- Did the project make sense to the people you work with?
  - What were your first impressions of the toolkit paper work?
  - What were your impressions of the team meetings – do you think they are a necessary part of the project?
- Do you think that the people you work with have a clear understanding of the role they play in implementing PRACTISE?
- Do the people you work with like the intervention?
- Do they think it is worthwhile implementing?

**Cognitive participation**

- Were there key people driving the implementation of PRACTISE after the initial project set-up meeting?
- Was there enough direction in getting going at the start of the project?
  - Was there anything in particular that helped to get going?
- Do you think there has there been sufficient involvement of other professions in implementing PRACTISE?
  - Has one profession taken the lead e.g. PTs, OTs, RAs, TIs?
- Were some people more on board than others?
- Did anything get in the way of implementing PRACTISE?
- Has using PRACTISE affected how your work is organised?
  - In what way e.g. changes to paperwork, time allocation?

**Collective action [Using PRACTISE]**

- Are the stroke therapy team able to implement PRACTISE?
- Can you describe in your own words how you use the intervention?
  - How do you choose patients for PRACTISE?
  - Can you tell us about how you provide exercises to patients?
  - How do you decide how many repetitions to prescribe?
  - How do you go about communicating the prescribed exercises?
  - Are there any ways that you have changed or adapted components of PRACTISE?
  - How has your work setting gone about organising any equipment needed for PRACTISE?
- Do you think your colleagues use PRACTISE in the same way that you do?
- How do you monitor how much exercise that patients are doing?
- Do you have a way of measuring if patients are improving?
- Do you make any recommendations to patients when they are discharged from your service?
- Have you encountered any problems using PRACTISE?
  - Are people confident that PRACTISE is being used as it should be?
  - Can you describe any ways in which the intervention has been adapted?
  - Do you feel like there has been enough support from your work setting to engage in the project and implement the intervention?
  - Is there anything in particular that supported the implementation of the intervention?

**Reflexive monitoring**

- - Do you think PRACTISE works?
  - Do the people you work with think it was worth being involved in the project?
  - Will you continue to use PRACTISE?
  - Would you/have you made any changes to the whole process based on your experiences?
    - - The way the team meetings were organised?
      - Refining the intervention?
